# Personalised Nutraceutical Treatment Guided by *MTHFR* Genotype in Mental Health: A Retrospective Cohort Study

**DOI:** 10.3390/nu18172791

**Published:** 2026-08-26

**Authors:** Cristina Beer, Fiona Rae, Mikayla Watt, Maciej Trzaskowski, Clarissa Yates, Annalese Semmler, Joanne Voisey

**Affiliations:** 1 Centre for Genomics and Personalised Health, School of Biomedical Sciences, Faculty of Health, Queensland University of Technology, Kelvin Grove, QLD 4059, Australia; cristina.beer@hdr.qut.edu.au (C.B.); f.rae@qut.edu.au (F.R.); 2 Cris Medical Services, Robina, QLD 4226, Australia; mikaylagwatt@gmail.com; 3 Ketim Technologies, Brisbane City, QLD 4000, Australia; maciej@ketim.com.au (M.T.); clarissa.yates@ketim.com.au (C.Y.); 4 School of Clinical Sciences, Faculty of Health, Queensland University of Technology, Kelvin Grove, QLD 4059, Australia; annalese.semmler@qut.edu.au

**Keywords:** *MTHFR*, one-carbon metabolism, L-methylfolate, nutraceuticals, personalised nutrition, psychological distress

## Abstract

Background & Aims: One-carbon metabolism plays a central role in neurotransmitter synthesis, methylation capacity, and neurobiological resilience. Variants in the methylenetetrahydrofolate reductase (*MTHFR*) gene can reduce enzymatic activity, affecting folate- and methionine-cycle functions and potentially influencing biological pathways relevant to mood and anxiety disorders. Personalised nutraceutical treatment strategies, particularly those addressing methylation capacity through targeted B-vitamin, folate, and adjunctive metabolic interventions are increasingly implemented in integrative clinical practice, yet evidence regarding their clinical outcomes remains limited. Methods: We conducted a retrospective cohort study of 50 adults attending an integrative general practice clinic for anxiety and/or depression. All received personalised nutraceutical treatment informed by clinical assessment, laboratory testing and, for 37/50 patients, *MTHFR* genotyping. Psychological distress was measured using the Kessler-10 (K10) scale at baseline and approximately three months later. Secondary analyses evaluated whether outcomes differed by *MTHFR* genotype, whether specific supplements (e.g., L-methylfolate and SAMe) were associated with greater improvement, whether biomarker changes correlated with symptom change, and the safety/tolerability profile. Results: Across the full cohort, mean K10 scores significantly decreased by four points over the treatment period, with 72% of patients showing clinical improvement. Reductions in psychological distress were seen across all *MTHFR* genotypes, including individuals with homozygous variant genotypes. Supplement-specific analyses showed improvement among those receiving methylfolate or SAMe, although the differences were not statistically significant. Following nutraceutical treatment, biomarker analyses demonstrated significant increases in serum vitamin B12 and modest reductions in homocysteine, but biomarker shifts did not correlate strongly with K10 change. No serious adverse events or clinically significant abnormalities in liver or renal function were identified. Conclusions: In this real-world primary care cohort, personalised nutraceutical treatment, grounded in one-carbon metabolism support and applied alongside usual care, was associated with clinically meaningful reductions in psychological distress. Outcomes were comparable across *MTHFR* genotypes when treatments were appropriately tailored, suggesting that genotype and biomarker-informed nutraceutical strategies may mitigate potential metabolic disadvantages. These findings support further controlled research into precision nutraceutical psychiatry for anxiety and depression. Secondary analyses of genotype subgroup, specific supplements, and biomarker–outcome associations are reported alongside Benjamini–Hochberg FDR-adjusted *p*-values and should be interpreted as hypothesis-generating.

## 1. Introduction

Anxiety and depressive disorders are among the leading contributors to global disease burden, with complex aetiologies involving interactions between genetic vulnerability, nutritional status, inflammation, neurotransmitter dynamics, and environmental stressors [1,2]. Despite advances in pharmacological and psychotherapeutic interventions, treatment-resistant or only partially responsive presentations remain common in primary care, prompting interest in personalised biological approaches that address underlying metabolic, genomic, and biochemical contributors to mental illness [3,4,5]. In particular, genetic variants that alter nutrient requirements or metabolic function provide an opportunity to tailor treatment to individual profiles, potentially improving outcomes across diverse patient groups.

One-carbon metabolism, which encompasses the folate, homocysteine, and methylation pathways, has gained substantial attention in psychiatric research due to its centrality in monoamine synthesis, epigenetic regulation, neuroplasticity, and cellular energy production [6,7,8,9]. At the centre of this network is the enzyme 5, 10-methylenetetrahydrofolate reductase (MTHFR), which converts 5,10-methylene-THF to 5-methyltetrahydrofolate (5-MTHF), the methyl donor required for the remethylation of homocysteine to methionine via methionine synthase [10,11]. This reaction generates S-adenosyl-L-methionine (SAMe), the universal methyl donor essential for the synthesis and regulation of dopamine, serotonin, norepinephrine, phosphatidylcholine, creatine, nitric oxide, and numerous neurobiological pathways implicated in mood regulation [8,9,10].

Two common *MTHFR* polymorphisms, c.665C>T (p.Ala222Val; rs1801133, formerly known as C677T) and c.1286A>C (p.Glu429Ala; rs1801131, formerly known as A1298C), are associated with reduced MTHFR enzyme activity [12,13,14]. The c.665T allele yields an alanine-to-valine substitution associated with reduced enzyme activity, particularly in homozygotes, and particularly under conditions of low riboflavin availability [14,15,16]. The c.1286A>C polymorphism affects a regulatory domain and produces a milder but clinically relevant reduction in activity, especially when present in compound heterozygosity with c.665C>T [12,13]. Carriers of these variants frequently exhibit higher homocysteine levels, lower folate status, reduced SAMe levels, impaired DNA methylation, and increased oxidative stress; these pathophysiological features have also been associated with mood disorders [13,14,17,18,19].

Studies have reported associations between *MTHFR* polymorphisms and a wide range of neuropsychiatric conditions, including major depressive disorder, schizophrenia, bipolar disorder, autism spectrum disorder, attention-deficit hyperactivity disorder, and cognitive impairment. However, findings vary by ethnicity, dietary folate intake, inflammatory load, and comorbid metabolic factors [2,8,20]. Meta-analyses indicate a small but significant increase in depression risk among homozygous c.665C>T carriers, particularly within Asian and European ancestry groups [21,22,23]. However, the functional consequences of impaired folate methylation may be more clinically relevant than genotype alone: individuals with high homocysteine levels, low SAMe levels, low folate/B12 status, or elevated whole-blood histamine often exhibit depressive, anxious, or agitated symptomatology regardless of *MTHFR* genotype [8,17,24]. This suggests biochemical markers may provide greater clinical utility than *MTHFR* genotype alone when assessing neuropsychiatric conditions and tailoring intervention.

The one-carbon cycle contributes methyl groups for the synthesis of serotonin, dopamine, and norepinephrine by supporting tetrahydrobiopterin (BH4) regeneration and SAMe-dependent methylation reactions [6,10,25]. BH4 is a critical cofactor for tryptophan hydroxylase and tyrosine hydroxylase, the rate limiting enzymes in monoamine synthesis. Impaired methylation may reduce BH4 availability, thereby dampening monoamine production, impairing stress resilience, and promoting depressive symptoms [25].

Elevated homocysteine levels have been associated with neurotoxicity through N-methyl-D-aspartate (NMDA) receptor overstimulation, increased oxidative stress, mitochondrial dysfunction, and vascular impairment [9,26,27]. Elevated homocysteine levels are also linked to structural brain changes, cognitive decline, and reduced hippocampal volume, further supporting its relevance to mood disorders [19,27,28]. Conversely, reducing homocysteine levels via B-vitamin supplementation improves methylation efficiency and may restore neurochemical balance in susceptible individuals [28,29,30].

Given the biological centrality of one-carbon metabolism in neural function, nutraceutical strategies that support methylation capacity have gained clinical and research attention [2,31]. L-methylfolate (5-MTHF) is the bioactive folate form that bypasses MTHFR and effectively increases central nervous system methyl-folate levels. It has demonstrated efficacy as an adjunctive antidepressant in several randomised controlled trials, including in treatment-resistant depression populations [32,33,34].

Vitamin B12 (methylcobalamin or hydroxocobalamin) is required for methionine synthase activity. Deficiency is strongly associated with depressive symptoms, cognitive impairment, and elevated homocysteine, and supplementation has shown antidepressant and cognitive benefits [27,35]. Vitamin B6 (pyridoxal-5-phosphate) is essential for transsulphuration pathways that convert homocysteine to cystathionine. Low levels correlate with anxiety, irritability, and reduced serotonin synthesis [36]. Riboflavin (B2) is a key cofactor for MTHFR. Riboflavin supplementation reduces homocysteine levels, particularly in homozygous c.665C>T individuals, and enhances MTHFR enzyme stability [14,16,37].

SAMe is an exogenous methyl donor with antidepressant efficacy comparable to conventional antidepressants in meta-analyses, particularly for anergia and treatment-resistant presentations [32,38,39,40]. Zinc and magnesium are involved in NMDA receptor regulation, GABAergic modulation, neuroinflammation, and HPA axis activity. Zinc deficiency is associated with treatment-resistant depression, and magnesium supplementation has demonstrated rapid antidepressant effects [41,42]. Omega-3 fatty acids (EPA and DHA) modulate inflammatory cytokines, membrane fluidity, serotonin receptor function, and neuroplasticity. EPA-rich formulations show consistent antidepressant augmentation effects [43]. Finally, evidence shows that N-acetylcysteine (NAC), a glutathione precursor, can improve depression, anxiety, addiction, and bipolar depression by modulating oxidative stress and glutamate dysregulation [44]. Collectively, these data have driven the emergence of nutritional psychiatry and precision nutraceutical medicine, wherein interventions are personalised based on genomic, metabolic, and biochemical profiles rather than a one-size-fits-all approach [4,5,45,46,47].

Efforts to characterise biological subtypes (“biotypes”) of mental illness have identified patterns of methylation abnormalities, inflammation, oxidative stress, kynurenine metabolism, mitochondrial inefficiency, and trace mineral imbalances [24]. Fryar-Williams and Strobel have shown that *MTHFR* genotypes correspond to distinct biochemical phenotypes, including hypermethylation-associated riboflavin depletion in homozygous c.665C>T individuals versus biochemical features suggestive of reduced methylation capacity, including elevated histamine levels and low B6 status in wild-type c.665C>T groups [24]. These phenotypes respond differently to targeted nutrient interventions, suggesting the value of personalised supplementation strategies.

In parallel, whole-blood histamine (a functional inverse marker of methylation), SAMe/SAH ratios, homocysteine levels, copper-to-zinc ratios, and oxidative stress markers provide additional mechanistic insight into individual symptom drivers and treatment targets [24]. Despite emerging mechanistic and clinical evidence, real-world data describing the effectiveness of integrated, genotype-guided nutraceutical protocols within primary care settings remain limited. Questions of clinical relevance include whether personalised nutraceutical treatments can reduce psychological distress, as measured using the Kessler Psychological Distress Scale (K10) in a heterogeneous primary care sample, and whether individuals with *MTHFR* risk genotypes respond differently to intervention. The potential impact of specific nutraceuticals, particularly L-methylfolate and SAMe, associated with superior outcomes, is also of interest, as is whether changes in key biomarkers (e.g., homocysteine, folate, B12, and histamine) correlate with symptom change. The tolerability and safety of this approach in real-world practice will be evaluated.

To address these questions, we conducted a retrospective analysis of 50 adults, with anxiety or depression who received care at an integrative general practice clinic. *MTHFR* genotype data were available for 37 participants. All patients received personalised nutraceutical treatment informed by biochemical and genetic data, providing an opportunity to explore whether a personalised approach can support improvement across diverse *MTHFR* genotypes in routine clinical practice.

## 2. Materials and Methods

### 2.1. Study Design and Setting

This study is a retrospective observational cohort analysis of routine clinical data collected at a single integrative general practice clinic on the Gold Coast, Australia, which provides combined conventional and nutritional medicine services for mental health. The clinic routinely assesses patients’ psychological, metabolic, nutritional, and genetic profiles and implements personalised nutraceutical interventions accordingly. All data were extracted from electronic clinical records and were fully de-identified before analysis.

Following baseline assessment, including administration of the K10 questionnaire, biomarker testing, and MTHFR genotyping, participants underwent an intervention for approximately three months (10–16 weeks). At the end of the intervention period, the K10 questionnaire and biomarker testing were re-administered to assess changes in psychological distress. Adherence was assessed clinically by patient self-report.

### 2.2. Cohort

Patients were included if they were over 18 years of age; held a Mental Health Care Plan (MHCP) indicating a primary diagnosis of anxiety and/or depressive symptoms; completed at least one follow-up visit with a paired Kessler Psychological Distress Scale (K10) measurement following commencement of nutraceutical treatment; and maintained stable psychotropic medication regimens, with no initiation or major dose changes during the treatment window. Patients were excluded if they had acute psychosis or unstable severe mental illness, missing baseline or follow-up K10 scores, and/or significant medication changes during treatment.

Fifty patients met the criteria and were included in the analysis. Among these, 42 (84%) were female; the mean age was 42.7 ± 14.2 years (range 18–78); 48 (96%) were Caucasian, 1 (2%) was Indian, and 1 (2%) was Brazilian; 26 (52%) were on stable psychotropic medications (predominantly selective serotonin reuptake inhibitors/serotonin-norepinephrine reuptake inhibitors [SSRI/SNRIs]); and 37 (74%) had *MTHFR* genotype results available.

### 2.3. Personalised Nutraceutical Intervention

The personalised nutraceutical protocol was designed individually for each patient based on clinical assessment, laboratory markers, dietary intake, and (where available) *MTHFR* genotype. Supplementary Table S1 provides an overview of each patient’s relevant biomarkers, *MTHFR* genotype if available, and prescribed treatment. Core treatment principles targeted one-carbon metabolism, methylation efficiency, neurotransmitter synthesis, and micronutrient optimisation. Personalised nutraceutical protocol was either compounded at a pharmacy or supplied as a commercial supplement, depending on the patient.

Common components included L-methylfolate, folinic acid, or 5-MTHF, provided through high-potency B-complex formulations or standalone products, used more frequently in individuals with *MTHFR* variants, elevated homocysteine levels, low folate status, or biomarkers suggestive of reduced methylation capacity. L-methylfolate was typically prescribed at 15 mg/day, based on prior efficacy data [32]. Vitamin B12 (methylcobalamin or hydroxocobalamin) was administered orally or intramuscularly; IM injections were used in those with deficiency, low-normal levels, or profound fatigue and cognitive symptoms. Vitamin B6 (P5P) was included in most B-complex preparations to support neurotransmitter synthesis and homocysteine metabolism. Riboflavin (B2) was included as part of B-complex formulations, with increased emphasis in known homozygous c.665C>T genotypes given riboflavin’s role in stabilising the thermolabile enzyme [15,37]. Dosages were adjusted based on clinical profile, lab markers (homocysteine, folate), and genotype where available (i.e., higher 5-MTHF dosing for heterozygous/homozygous c.665C>T variants), with adjustments made for baseline folate status and response.

SAMe (S-adenosylmethionine) was used in 7 patients for treatment-resistant depression. *MTHFR* c.665C>T genotypes, baseline biomarkers, and patient responses guided SAMe dosing. For example, individuals with heterozygous or homozygous c.665C>T genotypes typically received higher doses. Zinc and magnesium were prescribed for stress regulation, anxiety, sleep disturbance, reduced methylation capacity, or high copper-to-zinc ratios. Omega-3 fatty acids were typically provided as EPA-rich fish oil. Other adjunctive supports included vitamin D, herbal anxiolytics (e.g., Hypericum perforatum), probiotics, and progesterone (in perimenopausal women).

Although no formal written algorithm was in use at the time of clinical care because this represented routine integrative practice, treatment selection followed reproducible clinical decision rules linking genotype and biomarker findings to specific supplement classes and dose ranges. Specifically: (i) 5-methyltetrahydrofolate (5-MTHF, typically 15 mg/day) and riboflavin were prioritised in patients with c.665C>T variants and/or elevated homocysteine; (ii) intramuscular vitamin B12 was used where serum B12 was low-normal or fatigue was prominent; and (iii) SAMe was added in treatment-resistant presentations, with dose weighted upward in c.665C>T carriers. These rules were retrospectively documented and are reflected patient-by-patient in Supplementary Table S1, which lists each patient’s genotype, biomarkers, and prescribed supplements. We acknowledge that these were clinician-derived heuristics rather than a pre-registered algorithm and that this individualisation is inherent to real-world integrative practice.

### 2.4. Outcome Measures

#### 2.4.1. Primary Outcome: Psychological Distress (K10)

The Kessler Psychological Distress Scale (K10) was used to quantify symptom severity before and after approximately three months of intervention. The K10 is a validated 10-item questionnaire assessing anxiety and depressive symptoms over the previous four weeks. It is widely used in Australian primary care and population health [48]. Scores range from 10 to 15 indicating low psychological distress, 16 to 29 indicating moderate distress, and ≥30 indicating severe distress. The primary outcome was the change in K10 from baseline to first follow-up K10, with negative values indicating improvement.

#### 2.4.2. Secondary Outcomes

*MTHFR* genotype was assessed using PCR-based clinical genotyping of peripheral blood specimens for c.665C>T and c.1286A>C, which was available for 37 patients. *MTHFR* genotype results were extracted from commercial clinical laboratory reports. Genotyping was performed by the National Association of Testing Authorities (NATA)-accredited laboratory, Sullivan and Nicolaides Pathology, Bowen Hills, Queensland, Australia, using PCR-based clinical genotyping of peripheral blood specimens. As this was a retrospective clinical records study, the DNA extraction and PCR assays were performed by the commercial provider rather than the study team. For *MTHFR* variants, genotypes were categorised as wild-type (c.[665=];[665=], c.[1286=];[1286=]), heterozygous (c.[665=];[665C>T] or c.[1286=];[1286A>C]), homozygous variant (c.[665C>T];[665C>T] or c.[1286A>C];[1286A>C]), or compound heterozygous (c.[665=];[665C>T] and c.[1286=];[1286A>C]). Where available, paired baseline and follow-up biomarker measurements were extracted for serum/plasma homocysteine, serum folate, serum vitamin B12, whole-blood histamine, selected micronutrients (e.g., zinc and copper), and C-reactive protein. Due to individualised testing, sample sizes varied (e.g., homocysteine n = 30 paired; B12 n = 37; and folate n = 19).

### 2.5. Statistical Analysis

Statistical analyses were performed in R version 4.4.1 [49] using the base stats, car [50], effsize [51], and pwr [52] packages, with post hoc power and minimum detectable effect size (MDES) calculations cross-checked in G*Power 3.1.9.7 [53]. The primary outcome (change in K10 from baseline to follow-up) was tested with a paired t-test on the full cohort (n = 50) and effect size reported as Cohen’s dz with 95% confidence intervals. Secondary between-group comparisons of ΔK10 across MTHFR genotypes were conducted separately for the two loci: one-way ANOVAs on the c.665C>T three-level classification (wild-type, heterozygous, homozygous variant; n = 37) and, in a parallel analysis, on the c.1286A>C three-level classification (n = 37). Compound heterozygotes were retained in each single-locus ANOVA, reflecting their c.665C>T or c.1286A>C genotype at that locus; results were reproduced in a sensitivity analysis excluding compound heterozygotes. Because regression to the mean and unbalanced baseline severity are plausible alternative explanations for a null genotype effect, the primary genotype comparisons were re-run using ANCOVA with follow-up K10 as the outcome and baseline K10 as a covariate, using Type III sums of squares. Effect sizes for ANOVA/ANCOVA models are reported as partial η^2^ with 95% confidence intervals; for two-group supplement comparisons, Cohen’s d with 95% CIs is reported. Allele–dosage regression was performed with two models: (i) a main-effects model including T-count (c.665C>T alleles, 0–2) and C-count (c.1286A>C alleles, 0–2) as continuous predictors of ΔK10; and (ii) a secondary model additionally including a T-count × C-count interaction term to test whether compound genotype burden predicts outcome beyond additive allele effects. Full model output (e.g., unstandardised and standardised coefficients, standard errors, 95% CIs, individual predictor *p*-values, F-statistic, model *p*-value, and R^2^ and adjusted R^2^) was reported. Paired within-subject changes in homocysteine, serum folate, and vitamin B12 were tested with paired t-tests; histamine (n = 2 paired) was not analysable and is excluded from inferential analyses. Associations between biomarker change and K10 change were assessed with Pearson correlations. To address multiple testing across the secondary analysis families (e.g., genotype subgroup comparisons, supplement subgroup comparisons, biomarker change, and biomarker–K10 correlations), Benjamini–Hochberg false discovery rate (FDR) adjusted *p*-values are reported alongside nominal *p*-values, with FDR applied within each analysis family. Alpha was set at nominal *p* < 0.05, two-tailed. To assess the risk of informative missingness in the biomarker analyses, we compared baseline age, sex, baseline K10 scores, *MTHFR* genotype distribution, and psychotropic medication status between patients with vs. without paired follow-up biomarker data, separately for homocysteine, serum folate, and vitamin B12 (independent t-tests and Fisher’s exact tests; Supplementary Table S2). Post hoc minimum detectable effect sizes (α = 0.05, 80% power, two-tailed) were computed for the primary paired K10 change and for each key secondary comparison (e.g., c.665C>T ANOVA, c.1286A>C ANOVA, methylfolate vs. no-methylfolate, and SAMe vs. no-SAMe) to contextualise null findings as likely underpowered rather than as evidence of no effect. Given the exploratory, hypothesis-generating nature of the secondary analyses and the modest sample sizes, all secondary findings are interpreted as hypothesis-generating.

### 2.6. Ethics

All data were extracted from electronic clinical records and were fully de-identified before analysis. The Queensland University of Technology Human Research Ethics Committee approved the study (Project 6091).

## 3. Results

### 3.1. Patient Characteristics

The baseline characteristics of the cohort are summarised in Table 1. Key demographic and clinical findings include a mean baseline K10 score of 24.3 ± 10.3. Baseline distress levels (as measured by K10) were distributed as follows: low in 22% of patients, moderate in 22%, high in 30%, and very high in 26%.

Adherence to the personalised nutraceutical protocol was extracted from clinician notes at the follow-up visit and categorised as fully adherent, or partially adherent. In the present cohort, 45 patients ([90%]) were classified as fully adherent, and 5 ([10%]) as partially adherent. Biomarker changes at follow-up (particularly increases in serum vitamin B12) provided indirect physiological evidence of engagement with treatment in a subset of patients (see Section 3.5).

### 3.2. Primary Outcome: Change in Psychological Distress (K10)

The mean K10 score decreased from 24.28 ± 10.31 at baseline to 20.28 ± 8.39 at follow-up, demonstrating a mean reduction of 4.00 ± 5.74 points. A paired t-test indicated a statistically significant reduction, t(49) = 4.931, *p* < 0.0001, with a small-to-medium effect size (Cohen’s d = 0.388).

### 3.3. K10 Outcomes by MTHFR Genotype

A reduction in K10 scores was observed across all *MTHFR* genotype groups (see Table 2).

Full model output (e.g., unstandardised and standardised coefficients, standard errors, 95% CIs, individual predictor *p*-values, F-statistic, model *p*-value, and R^2^ and adjusted R^2^) is reported in Table 3.

Improvement in K10 scores did not vary meaningfully by c.665C>T genotype. A one-way ANOVA showed no statistically significant difference between groups (F(2,34) = 0.666, *p* = 0.5206).

To investigate the extended genotype model, a full seven-group genotype model (e.g., wild-type, heterozygous, homozygous variants, compound heterozygous, and double homozygous) showed no significant genotype effect, Global ANOVA *p* = 0.849.

The one-way ANOVA reported above used only the c.665C>T three-level classification (e.g., wild-type, heterozygous, and homozygous variant) as the grouping variable and included all 37 genotyped patients; compound heterozygotes were retained in whichever c.665C>T category their genotype placed them in. A parallel one-way ANOVA on the c.1286A>C three-level classification (n = 37) yielded F(2,34) = 0.507, *p* = 0.607. To adjust for possible differences in baseline distress severity across genotype groups, ANCOVA models with follow-up K10 as the outcome and baseline K10 as the covariate were fitted for both loci; results are reported in Table 2 (Panels A and B). Partial η^2^ with 95% confidence intervals is reported for each ANOVA model. Within-group Cohen’s dz with 95% CI is reported for each genotype subgroup in Table 2. The ‘No *MTHFR* genotype determined’ subgroup (n = 13) is presented in Table 2 as a reference row outside the between-group statistical panels.

### 3.4. Supplement-Specific Effects

Patients who received methylfolate (n = 21, −5.24 ± 6.57) demonstrated greater improvement in K10 scores compared to those who did not receive methylfolate (n = 29, −3.10 ± 4.97). However, this difference was not statistically significant, *p* = 0.197. Similarly, patients who received SAMe (n = 7, −5.14 ± 2.97) showed greater improvement in K10 scores than those who did not receive SAMe supplementation (n = 43, −3.81 ± 6.07). However, no statistically significant difference was found, *p* = 0.575.

An exploratory multi-supplement ANOVA examining nine supplement pattern groups found no significant difference between groups (*p* = 0.877). Overall, methylfolate and SAMe users showed numerically greater improvement; however, the sample sizes limited significance.

For the supplement-specific subgroup analyses, Cohen’s d with 95% confidence intervals is reported for methylfolate vs. no methylfolate: d = −0.283 [−0.859, 0.293], and SAMe vs. no SAMe: d = −0.230 [−1.051, 0.591]. Benjamini–Hochberg FDR-adjusted *p*-values, computed within the supplement-comparison analysis family, are reported alongside nominal *p*-values for the methylfolate comparison (nominal *p* = 0.197; FDR-adjusted *p* = 0.591), the SAMe comparison (nominal *p* = 0.575; FDR-adjusted *p* = 0.863), and the nine-group ANOVA (nominal *p* = 0.877; FDR-adjusted *p* = 0.877). Partial η^2^ with 95% CI is reported for the nine-group ANOVA. All secondary supplement comparisons are framed as hypothesis-generating.

### 3.5. Biomarker Changes

Among patients with paired homocysteine results (n = 30), a mean reduction in levels was observed (−0.91 ± 3.11 µmol/L); however, this finding was not statistically significant. Additionally, no statistically significant correlation was found between changes in homocysteine and changes in K10 scores, r = 0.119, *p* = 0.532.

For vitamin B12 (n = 37 paired), levels increased significantly at follow up, +104.78 ± 279.97 pmol/L, *p* = 0.029. No statistically significant correlation was identified between changes in vitamin B12 levels and K10 improvement, r = −0.017, *p* = 0.923. No significant change was found in serum folate (n = 19 paired) levels at follow up, and no significant correlation was found with K10 score changes, r = 0.177, *p* = 0.468. Histamine (n = 2 paired) was not analysable due to the limited sample size of patients who retested this biomarker. Overall, vitamin B12 levels improved robustly while other biomarker changes did not appear to have an association with changes in psychological symptoms.

Paired whole-blood histamine was available for only 2 patients and was therefore not analysed as a correlation with K10 change.. To assess whether missingness in the paired biomarker data was informative, a sensitivity analysis compared baseline characteristics (e.g., age, sex, baseline K10, baseline biomarker value where available, MTHFR genotype distribution, and psychotropic medication status) between patients with vs. without paired follow-up data for homocysteine, serum folate, and vitamin B12. The results are shown in Supplementary Table S2. Sensitivity analyses comparing patients with vs. without paired follow-up biomarker data showed the following baseline differences reaching nominal significance: Homocysteine – sex (Fisher *p* = 0.033). All other comparisons were non-significant. These differences are modest in magnitude and do not indicate systematic informative missingness. Benjamini–Hochberg FDR-adjusted *p*-values are reported alongside nominal *p*-values for the four biomarker–K10 correlation analyses.

### 3.6. Safety

No significant changes in ALT, AST, GGT, creatinine, or eGFR levels were found. There were no supplement-induced severe adverse events. Mild transient effects were reported in some cases, such as headaches, restlessness, or insomnia, and were resolved following dose adjustments. No cases of hypomania/mania, psychosis, or allergic reactions were observed.

## 4. Discussion

This retrospective cohort study evaluated real-world outcomes following personalised nutraceutical treatment among 50 adults with anxiety and/or depressive symptoms in a primary-care setting. The intervention targeted one-carbon metabolism, methylation capacity, micronutrient optimisation, and related biochemical pathways. We observed a statistically significant and clinically meaningful reduction in psychological distress (mean ΔK10 = −4.0 points), with 72% of patients demonstrating improvement. Importantly, despite differences in *MTHFR* genotype, clinical response was consistent across wild-type, heterozygous, and homozygous variant groups. Given that treatment was personalised according to genotype, this uniform improvement suggests that tailoring nutraceutical interventions to an individual’s *MTHFR* status may be a clinically useful strategy; however, controlled studies are required to determine whether this approach produces comparable benefit across genotype groups. Supplement-specific effects showed numerically greater improvement among users of L-methylfolate and SAMe but did not reach statistical significance due to small subgroup sizes. Biomarker shifts confirmed physiological engagement, especially B12 repletion, but did not correlate directly with K10 change. Overall, the personalised nutraceutical protocol appeared safe and well-tolerated. The findings are consistent with a growing body of evidence supporting the role of targeted nutritional and metabolic interventions in mental health treatment [32,45,54].

One-carbon metabolism supports essential neurobiological functions, including DNA and histone methylation, monoamine neurotransmitter synthesis, phospholipid metabolism, membrane fluidity, and cellular antioxidant capacity [6,7,10]. Impairments in this pathway, whether due to genetic polymorphisms (e.g., *MTHFR* variants), nutritional deficiencies (folate, B12, B6, riboflavin), elevated homocysteine, oxidative stress, or inflammation can result in decreased SAMe availability, impaired neurotransmitter synthesis, and increased neurotoxic stress [7,26,27,28].

Elevated homocysteine levels have been linked to NMDA receptor overstimulation, oxidative damage, endothelial dysfunction, and hippocampal atrophy, collectively contributing to affective symptomatology and cognitive impairment [19,26,27,28]. Lower SAMe levels, observed in individuals with folate/B12 deficiency or MTHFR impairment, negatively impact methylation reactions essential for serotonin, dopamine, and norepinephrine regulation [25]. These pathways align with core neurobiological models of depression involving monoaminergic dysregulation, impaired neuroplasticity, mitochondrial dysfunction, and neuroinflammation [6,7,27]. Given these mechanistic intersections, nutritional deficiencies or genetic constraints affecting one-carbon metabolism may play a contributory role in mood disorders, particularly in treatment-resistant or partially responsive presentations [32].

A central aim of this study was to determine whether personalised nutraceutical treatment informed by *MTHFR* genotype and biochemical markers was associated with symptom improvement, and whether treatment response differed across genotype groups. The absence of significant genotype effects across c.665C>T, c.1286A>C, compound heterozygotes, and at-risk homozygotes has several important implications. First, MTHFR genotype may influence nutritional requirements and metabolic vulnerability, but it should not be interpreted as a deterministic predictor of psychiatric symptoms or treatment response. Although MTHFR polymorphisms reduce enzyme activity and can impair methylation, genotype alone does not reliably predict mood symptom severity in cross-sectional psychiatric studies [22,23]. Instead, genotype appears to influence the physiological requirements for adequate folate metabolism. For example, as observed in our cohort, homozygous c.665C>T individuals show lower serum and red-cell folate levels for the same dietary intake, greater sensitivity to folate deficiency, reduced ability to generate 5-MTHF, and higher homocysteine levels, particularly with riboflavin insufficiency [15,16,17,37]. However, when these vulnerabilities are addressed with L-methylfolate, riboflavin, and cofactor support, the expected biochemical disadvantages can often be mitigated [16,29,37]. These findings are consistent with the possibility that targeted nutraceutical strategies (e.g., higher 5-MTHF dosing, inclusion of riboflavin, and B12 injections) may reduce genotype-associated metabolic disadvantage; however, controlled studies are required to test this directly.

Additionally, our findings align with “precision rescue” models emerging in psychiatric genomics, where genotype-informed biochemical support normalises clinical outcomes across genotypes [24,32]. Analogous examples include CYP450-guided psychotropic dose adjustments improving tolerability, folate-cycle supplementation reducing neural tube defect risk in *MTHFR* carriers, and riboflavin supplementation normalising blood pressure or homocysteine in homozygous c.665C>T individuals [16].

By bypassing the metabolic bottleneck (e.g., providing 5-MTHF instead of folic acid) and supporting cofactor demands, genotype became less predictive of outcome because the treatment was specifically designed to address genotype-induced impairments. This aligns with work by Fryar-Williams and colleagues showing that different *MTHFR* genotypes correspond to distinct biochemical phenotypes, some characterised by hypermethylation patterns (c.665C>T homozygous) and others by reduced methylation capacity (c.665C>T wild-type), with each requiring different nutrient combinations for optimal correction [54].

It is important, however, to note that the ‘No *MTHFR* genotype determined’ subgroup (n = 13) improved to a similar degree (ΔK10 = −3.46) as the genotyped subgroups, despite receiving no genotype-informed dosing adjustment for *MTHFR*-specific variants. This observation does not adjudicate between competing explanations for the overall improvement and is equally consistent with (a) a non-specific benefit of general integrative clinical care independent of genotype-tailoring, (b) regression to the mean and natural history operating on a moderately distressed cohort with baseline mean K10 = 24.3, or (c) successful genotype-informed rescue of metabolic disadvantage. The current design cannot distinguish between these possibilities. Consistent with this, allele–dosage regression explained only 3.3% of the variance in ΔK10, and no genotype effect was detected in either the c.665C>T or c.1286A>C three-level ANOVAs. We therefore reframe the study’s secondary genotype findings as hypothesis-generating rather than as evidence of a precision-rescue mechanism, and we note that regression to the mean is a strong alternative explanation for the null between-genotype effect. Because baseline K10 distributions are broadly comparable across genotype groups, regression to the mean would be expected to operate similarly across groups and therefore predicts the observed absence of a genotype effect independent of any personalisation mechanism.

In addition, allele–dosage regression showed that combined c.665C>T and c.1286A>C allele counts explained only 3.3% of the variance in K10 change, which reflects several points. K10 is influenced by multiple axes (e.g., psychosocial stress, trauma, sleep, inflammation, and nutrition), which collectively dominate over genetic signal. Personalised methylation-targeted support may have reduced observable genotype-related differences in treatment response. *MTHFR* polymorphisms exert modest effect sizes in psychiatric conditions, even in large meta-analyses [22,23]. Furthermore, treatment response in psychiatry is multifactorial and rarely driven by any single variant. These findings support consensus recommendations that *MTHFR* variants should be interpreted as modifiable risk factors, not deterministic predictors of psychiatric outcomes [21].

Integrative nutraceutical treatment may represent a biologically rational adjunct to usual care. The observed improvement in psychological distress aligns with clinical and mechanistic evidence for several nutrient interventions included in the personalised protocols.

Randomised controlled trials have demonstrated that L-methylfolate (15 mg/day) significantly improves depressive symptoms when added to SSRI therapy, particularly among individuals with elevated inflammatory markers, low folate status, or treatment-resistant profiles [32]. Its ability to bypass MTHFR and enhance methylation makes it uniquely suitable for patients with reduced folate-cycle efficiency. In our cohort, the methylfolate group demonstrated numerically greater symptom reduction (−5.24 points) than the non-methylfolate group (−3.10 points). While not statistically significant due to sample size constraints, this pattern mirrors RCT findings and supports biological plausibility.

Robust evidence supports the efficacy of SAMe as an antidepressant, with meta-analyses showing comparable efficacy to tricyclic antidepressants and SSRIs, and particular benefits for low energy, anergia, and melancholic features [55]. Its direct contribution to methylation and monoamine synthesis makes it relevant for patients with low SAMe or elevated SAH levels. Although only seven patients received SAMe, their mean improvement (−5.14 points) was again numerically larger than the cohort average.

Vitamin B12 deficiency is associated with depressive symptoms, cognitive impairment, fatigue, and elevated homocysteine [35]. Supplementation improves mood, especially in B12-deficient individuals or those with borderline levels [29,35]. B6 (P5P) is necessary for neurotransmitter synthesis (e.g., GABA, serotonin, and dopamine) and homocysteine transsulphuration [36]. Riboflavin stabilises the *MTHFR* enzyme and significantly lowers homocysteine in c.665C>T homozygotes [15,16,37]. Our biomarker data showed significant B12 repletion in the sample. Homocysteine declined modestly, consistent with expected biochemical correction. Although NAC was not widely used in this cohort, its mechanistic relevance is notable: NAC restores glutathione levels, modulates glutamatergic signalling, and reduces oxidative stress, leading to antidepressant and anxiolytic effects in several RCTs [44].

Importantly, the personalised nutraceutical protocol demonstrated excellent safety. No significant changes in liver or kidney function were observed, and no serious adverse events occurred. Mild transient effects (e.g., headaches, restlessness, and insomnia) resolved with dose adjustments. Concerns that high-dose methyl donors (L-methylfolate, SAMe) may precipitate agitation or hypomania in susceptible individuals were not borne out in this study, consistent with recent controlled studies showing low risk when used appropriately [32,50]. This suggests that nutraceutical psychiatry can be safely implemented in primary care with careful monitoring and adequate dosage control.

In the context of conventional psychiatry, despite widespread use of SSRIs and SNRIs, remission rates remain modest, and up to 30–50% of patients fail to respond adequately to first-line treatments [1,3]. Augmentation strategies are increasingly emphasised in guidelines, including psychotherapy, atypical antipsychotics, thyroid hormone, lithium, and evidence-based nutraceuticals [32]. Our findings support the inclusion of methylation-targeted nutraceutical augmentation as a low-risk, biologically grounded option, consistent with emerging precision psychiatry frameworks [45].

The nutritional psychiatry evidence base supports the use of folate/L-methylfolate augmentation [32], omega-3 fatty acids [43], zinc and magnesium [41,42], NAC for mood and anxiety [44], and B-vitamin complexes [29,35,36]. The present study adds real-world evidence supporting a multi-target, personalised approach rather than single-agent interventions.

*MTHFR* testing guidelines remain divided. Some consider *MTHFR* testing unnecessary for depression treatment due to small effect sizes [45]. Others suggest that testing may be useful for guiding folate form selection (e.g., 5-MTHF vs. folic acid), especially in treatment-resistant cases or where homocysteine levels are elevated [6,10,32].

Our findings support a balanced perspective in which genotype should not be used as a diagnostic or prognostic tool. However, genotype may guide treatment personalisation, particularly nutrient selection and cofactor support. Importantly, when addressed appropriately, individuals with risk genotypes respond similarly to others.

This study demonstrates several strengths, including the use of real-world data reflecting actual primary care practice, the inclusion of both genetic and biochemical information, the execution of an independent external statistical analysis to improve rigor, the use of a validated outcome measure (K10) and the integration of multi-nutrient personalised protocols..

Nevertheless, several limitations should be considered. The retrospective design prohibits causal inference, and there was no control group; therefore, regression to the mean, placebo effects, or benefits from ongoing psychotherapy cannot be excluded. Additionally, the small sample and small genotype subgroups reduce statistical power, and the heterogeneity of nutritional protocols introduces complexity. Biomarker retesting was incomplete (particularly histamine) and K10 captures overall distress but not specific symptom domains (e.g., anhedonia and sleep disturbance). Post hoc power/minimum detectable effect size (MDES) analyses were performed for key comparisons. Null findings in the smaller subgroup comparisons should therefore be interpreted as consistent with the study being under-powered to detect small-to-moderate effects.

Future research should include randomised controlled trials comparing personalised nutraceutical care with standard care. Furthermore, stratified designs comparing responders with non-responders by biochemical phenotype are recommended. Integration of advanced biomarkers (e.g., SAM/SAH ratios, BH4 levels, and metabolomics) could provide additional mechanistic insight. Longitudinal assessment of functional outcomes (e.g., work capacity, sleep, and quality of life) and examination of cost-effectiveness and quality-adjusted life years (QALYs) are also important. For clearer evidence of the robustness of the “treatment personalised to genotype” effect, future studies with larger sample sizes could include a standardised, non-personalised treatment comparator across genotypes to determine if changes in symptoms over time show a stronger difference than the equalised effect shown in the current cohort.

## 5. Conclusions

This retrospective cohort analysis provides real-world evidence that personalised nutraceutical treatment targeting methylation, one-carbon metabolism, and related biochemical pathways can reduce psychological distress in adults receiving primary-care mental health support. Despite small sample sizes, the direction of all effect sizes was in line with the literature and the average symptom reduction was clinically meaningful, with most patients benefiting.

Notably, *MTHFR* genotype did not predict treatment response, reinforcing that genotype should not be interpreted deterministically. Instead, when methylation vulnerabilities are identified and actively addressed with targeted nutrients, such as L-methylfolate, B12, B6, riboflavin, SAMe, zinc, magnesium, and omega-3 fatty acids, improvements in psychological distress were observed across *MTHFR* genotypes. These findings support the integration of nutraceutical psychiatry within precision mental health frameworks and highlight the promise of biochemical personalisation in improving outcomes for anxiety and depression. Prospective trials are warranted to confirm the magnitude of benefit and identify optimal treatment algorithms. All secondary genotype and supplement-specific findings should be interpreted as hypothesis-generating pending confirmation in prospective controlled studies.

## Figures and Tables

**Table 1:** Baseline characteristics of the cohort (n = 50).

Variable	Value
Age (years)	42.7 ± 14.2
Female	42 (84%)
Baseline K10	24.3 ± 10.3
Severe K10 ≥30	13 (26%)
On stable psychotropic medication	26 (52%)
Homocysteine (µmol/L) baseline	10.97 ± 3.33
Serum folate (nmol/L) baseline	31.55 ± 8.67
Vitamin B12 (pmol/L) baseline	426.84 ± 232.38
Whole-blood histamine (µg/L) baseline	49 ± 36 (n = 14)
*MTHFR* Genotypes (n = 37)	
c.[665=];[665=]	9 (24.3%)
c.[665=];[665C>T]	24 (64.9%)
c.[665C>T];[665C>T]	4 (10.8%)
c.[1286=];[1286=]	18 (43.2%)
c.[1286=];[1286A>C]	16 (43.2%)
c.[1286A>C];[1286A>C]	5 (13.5%)
- Compound heterozygotes	12 (32.4%)
- At-risk homozygotes (c.[665C>T];[665C>T] and/or c.[1286A>C];[1286A>C])	9 (24.3%)

Values are mean ± standard deviation (SD). Values adapted from analysis and clinical records.

**Table 2:** Mean K10 change in *MTHFR* genotype groups.

Genotype	n	Baseline K10 (Mean ± SD)	ΔK10 (Mean ± SD)	Cohen’s dz [95% CI]	Within-Group *p* (Nominal/FDR)
Panel A. c.665C>T classification (n = 37)					
c.[665=];[665=] (wild-type)	9	23.33 ± 10.26	−5.89 ± 4.04	−1.456 [−2.559, −0.353]	0.0024/0.0072
c.[665=];[665C>T] (heterozygous)	24	24.12 ± 11.00	−3.54 ± 5.74	−0.617 [−1.078, −0.156]	0.0061/0.0091
c.[665C>T];[665C>T] (homozygous variant)	4	23.00 ± 10.23	−4.25 ± 3.30	−1.286 [−3.437, 0.865]	0.0823/0.0823
Between-group ANOVA (ΔK10 ~ c.665C>T)			F(2,34) = 0.666	partial η^2^ = 0.0377 [0.0000, 0.1791]	*p* = 0.5206/n/a
ANCOVA (follow-up K10 ~ c.665C>T + baseline K10)			F(2,33) = 1.130	partial η^2^ = 0.0641 [0.0000, 0.2278]	*p* = 0.335/n/a
Panel B. c.1286A>C classification (n = 37)					
c.[1286=];[1286=] (wild-type)	18	23.78 ± 11.80	−4.17 ± 6.09	−0.684 [−1.237, −0.132]	0.0099/0.0149
c.[1286=];[1286A>C] (heterozygous)	16	23.94 ± 10.10	−3.69 ± 4.17	−0.883 [−1.512, −0.255]	0.0030/0.0090
c.[1286A>C];[1286A>C] (homozygous variant)	3	23.33 ± 5.03	−7.00 ± 4.36	−1.606 [−5.365, 2.153]	0.1086/0.1086
Between-group ANOVA (ΔK10 ~ c.1286A>C)			F(2,34) = 0.507	partial η^2^ = 0.0290 [0.0000, 0.1600]	*p* = 0.607/n/a
ANCOVA (follow-up K10 ~ c.1286A>C + baseline K10)			F(2,33) = 0.781	partial η^2^ = 0.0452 [0.0000, 0.1956]	*p* = 0.466/n/a
Panel C. Derived groupings (descriptive only—overlap with Panels A & B)					
Compound heterozygous (c.665C>T het AND c.1286A>C het)	12	23.00 ± 9.03	−2.50 ± 3.71	−0.675 [−1.379, 0.029]	0.0394/n/a
At-risk homozygous (c.665C>T homo and/or c.1286A>C homo)	9	23.14 ± 7.80	−5.43 ± 3.74	−1.453 [−2.779, −0.127]	0.0085/n/a
Reference (not in ANOVA)					
No *MTHFR* genotype determined	13	25.62 ± 10.13	−3.46 ± 7.36	−0.471 [−1.107, 0.166]	0.115/n/a

Values are mean ± SD unless otherwise stated. Panels A and B each include all 37 genotyped patients; individual patients contribute one row to each panel according to their genotype at each locus. Panel C shows derived groupings (compound heterozygotes; at-risk homozygotes) that overlap with Panels A and B; these rows are descriptive and were not included in the between-group ANOVAs. Per-row *p*-values are within-group paired t-tests (nominal/Benjamini–Hochberg FDR-adjusted within the genotype-comparison family). Between-group statistics are shown in the highlighted footer rows of each panel. The ‘No MTHFR genotype determined’ subgroup did not contribute to any between-group ANOVA and is reported for descriptive comparison only.

**Table 3:** Allele–dosage linear regression of ΔK10 on *MTHFR* T-count (c.665C>T alleles, 0–2) and C-count (c.1286A>C alleles, 0–2); n = 37.

Predictor	β (Unstd.)	SE	95% CI	β (Std.)	*p*
Model 1—main effects (ΔK10 ~ T-count + C-count)					
Intercept	−5.220	2.328	[−9.951, −0.490]	—	0.032
T-count (c.665C>T)	1.208	1.751	[−2.349, 4.766]	0.137	0.495
C-count (c.1286A>C)	−0.023	1.591	[−3.257, 3.210]	−0.003	0.988
Model 1 fit	F(2,34) = 0.333			R^2^ = 0.033; adj. R^2^ = −0.038	Model *p* = 0.719
Model 2—with T × C interaction (ΔK10 ~ T-count + C-count + T × C)					
Intercept	−3.820	2.429	[−8.761, 1.121]	—	0.125
T-count	−0.450	1.987	[−4.493, 3.593]	−0.051	0.822
C-count	−2.050	1.987	[−6.093, 1.993]	−0.256	0.310
T × C interaction	3.820	2.336	[−0.934, 8.574]	0.351	0.112
Model 2 fit	F(3,33) = 1.124			R^2^ = 0.093; adj. R^2^ = 0.010	Model *p* = 0.354

Model 1 fits ΔK10 as an additive function of T-count and C-count. Model 2 adds a T × C interaction term to test whether compound genotype burden (i.e., simultaneously carrying c.665C>T and c.1286A>C variant alleles) predicts outcome beyond additive allele effects. Standardised β allows comparison of the relative influence of the two loci.

## Data Availability

The raw data supporting the conclusions of this article will be made available by the authors upon request.

## Supplementary Materials

The following supporting information can be downloaded at: https://www.mdpi.com/article/10.3390/nu18172791/s1, Table S1: Patient *MTHFR* genotype, baseline biomarker profile, and treatment/supplement regimen; Table S2: Missingness sensitivity analysis for paired biomarker follow-up.

## References

[1] GBD 2019 Mental Disorders Collaborators (2022). Global, regional, and national burden of 12 mental disorders in 204 countries and territories, 1990–2019: A systematic analysis for the Global Burden of Disease Study 2019. Lancet Psychiatry.

[2] Sarris J., Murphy J., Mischoulon D., Papakostas G.I., Fava M., Berk M., Ng C.H. (2016). Adjunctive nutraceuticals for depression: A systematic review and meta-analyses. Am. J. Psychiatry.

[3] Gaynes B.N. (2009). Identifying difficult-to-treat depression: Differential diagnosis, subtypes, and comorbidities: Differential diagnosis, subtypes, and comorbidities. J. Clin. Psychiatry.

[4] Rosenblat J.D., Lee Y., McIntyre R.S. (2018). The effect of pharmacogenomic testing on response and remission rates in the acute treatment of major depressive disorder: A meta-analysis. J. Affect. Disord..

[5] Panagiotou N., Sagonas A., Ntoumou E., Salata E., Fotis T. (2025). Pharmacogenetic-guided treatment in major depressive disorder. World Acad. Sci. J..

[6] Bottiglieri T. (1996). Folate, vitamin B12, and neuropsychiatric disorders. Nutr. Rev..

[7] Kruman I.I., Mouton P.R., Emokpae R., Cutler R.G., Mattson M.P. (2005). Folate deficiency inhibits proliferation of adult hippocampal progenitors. Neuroreport.

[8] Papakostas G.I., Cassiello C.F., Iovieno N. (2012). Folates and S-adenosylmethionine for major depressive disorder. Can. J. Psychiatry.

[9] Mischoulon D., Fava M. (2002). Role of S-adenosyl-l-methionine in the treatment of depression: A review of the evidence. Am. J. Clin. Nutr..

[10] Froese D.S., Fowler B., Baumgartner M.R. (2019). Vitamin B12, folate, and the methionine remethylation cycle-biochemistry, pathways, and regulation. J. Inherit. Metab. Dis..

[11] Friso S., Choi S.-W. (2002). Gene-nutrient interactions and DNA methylation. J. Nutr..

[12] Weisberg I., Tran P., Christensen B., Sibani S., Rozen R. (1998). A second genetic polymorphism in methylenetetrahydrofolate reductase (MTHFR) associated with decreased enzyme activity. Mol. Genet. Metab..

[13] Lipp M., Pasternak A., Ward K. (2020). Impact of the MTHFR C677T genetic variant on depression. Curr. Psychiatry.

[14] Stengler M. (2021). The role of folate and MTHFR polymorphisms in the treatment of depression. Altern. Ther. Health Med..

[15] Yamada K., Chen Z., Rozen R., Matthews R.G. (2001). Effects of common polymorphisms on the properties of recombinant human methylenetetrahydrofolate reductase. Proc. Natl. Acad. Sci. USA.

[16] McNulty H., Strain J.J., Ward M. (2014). Riboflavin lowers blood pressure in hypertensive people with the MTHFR677TT genotype. Arch. Public Health.

[17] Bjelland I., Tell G.S., Vollset S.E., Refsum H., Ueland P.M. (2003). Folate, vitamin B12, homocysteine, and the MTHFR 677C→T polymorphism in anxiety and depression: The Hordaland Homocysteine Study: The Hordaland Homocysteine Study. Arch. Gen. Psychiatry.

[18] Murakami K., Mizoue T., Sasaki S., Ohta M., Sato M., Matsushita Y., Mishima N. (2008). Dietary intake of folate, other B vitamins, and omega-3 polyunsaturated fatty acids in relation to depressive symptoms in Japanese adults. Nutrition.

[19] Ford A.H., Flicker L., Singh U., Hirani V., Almeida O.P. (2013). Homocysteine, depression and cognitive function in older adults. J. Affect. Disord..

[20] Cuomo A., Beccarini Crescenzi B., Bolognesi S., Goracci A., Koukouna D., Rossi R., Fagiolini A. (2020). S-Adenosylmethionine (SAMe) in major depressive disorder (MDD): A clinician-oriented systematic review. Ann. Gen. Psychiatry.

[21] Araszkiewicz A.F., Jańczak K., Wójcik P., Białecki B., Kubiak S., Szczechowski M., Januszkiewicz-Lewandowska D. (2025). MTHFR gene polymorphisms: A single gene with wide-ranging clinical implications—A review. Genes.

[22] Wu Y.-L., Ding X.-X., Sun Y.-H., Yang H.-Y., Chen J., Zhao X., Jiang Y.-H., Lv X.-L., Wu Z.-Q. (2013). Association between MTHFR C677T polymorphism and depression: An updated meta-analysis of 26 studies. Prog. Neuro-Psychopharmacol. Biol. Psychiatry.

[23] Peerbooms O.L.J., van Os J., Drukker M., Kenis G., Hoogveld L., De Hert M., Delespaul P., Van Winkel R., Rutten B.P.F., MTHFR in Psychiatry Group (2011). Meta-analysis of MTHFR gene variants in schizophrenia, bipolar disorder and unipolar depressive disorder: Evidence for a common genetic vulnerability?. Brain Behav. Immun..

[24] Fryar-Williams S., Strobel J.E. (2015). Biomarkers of a five-domain translational substrate for schizophrenia and schizoaffective psychosis. Biomark. Res..

[25] Trefz F., Lichtenberger O., Blau N., Muntau A.C., Feillet F., Bélanger-Quintana A., van Spronsen F., Munafo A. (2015). Tetrahydrobiopterin (BH4) responsiveness in neonates with hyperphenylalaninemia: A semi-mechanistically-based, nonlinear mixed-effect modeling. Mol. Genet. Metab..

[26] Ho P.I., Collins S.C., Dhitavat S., Ortiz D., Ashline D., Rogers E., Shea T.B. (2001). Homocysteine potentiates beta-amyloid neurotoxicity: Role of oxidative stress. J. Neurochem..

[27] Smith A.D., Refsum H. (2016). Homocysteine, B vitamins, and cognitive impairment. Annu. Rev. Nutr..

[28] Sachdev P.S. (2005). Homocysteine and brain atrophy. Prog. Neuro-Psychopharmacol. Biol. Psychiatry.

[29] Almeida O.P., Marsh K., Alfonso H., Flicker L., Davis T.M.E., Hankey G.J. (2010). B-vitamins reduce the long-term risk of depression after stroke: The VITATOPS-DEP trial. Ann. Neurol..

[30] Gao S., Khalid A., Amini-Salehi E., Radkhah N., Jamilian P., Badpeyma M., Zarezadeh M. (2024). Folate supplementation as a beneficial add-on treatment in relieving depressive symptoms: A meta-analysis of meta-analyses. Food Sci. Nutr..

[31] Schefft C., Kilarski L.L., Bschor T., Köhler S. (2017). Efficacy of adding nutritional supplements in unipolar depression: A systematic review and meta-analysis. Eur. Neuropsychopharmacol..

[32] Papakostas G.I., Shelton R.C., Zajecka J.M., Etemad B., Rickels K., Clain A., Baer L., Dalton E.D., Sacco G.R., Schoenfeld D. (2012). L-methylfolate as adjunctive therapy for SSRI-resistant major depression: Results of two randomized, double-blind, parallel-sequential trials. Am. J. Psychiatry.

[33] Maruf A.A., Poweleit E.A., Brown L.C., Strawn J.R., Bousman C.A. (2022). Systematic review and meta-analysis of L-methylfolate augmentation in depressive disorders. Pharmacopsychiatry.

[34] Zajecka J.M., Fava M., Shelton R.C., Barrentine L.W., Young P., Papakostas G.I. (2016). Long-term efficacy, safety, and tolerability of L-methylfolate calcium 15 mg as adjunctive therapy with selective serotonin reuptake inhibitors: A 12-month, open-label study following a placebo-controlled acute study: A 12-month, open-label study following a placebo-controlled acute study. J. Clin. Psychiatry.

[35] O’Leary F., Samman S. (2010). Vitamin B12 in health and disease. Nutrients.

[36] Dakshinamurti K., Paulose C.S., Viswanathan M., Siow Y.L., Sharma S.K., Bolster B. (1990). Neurobiology of pyridoxine. Ann. N. Y. Acad. Sci..

[37] McAuley E., McNulty H., Hughes C., Strain J.J., Ward M. (2016). Riboflavin status, MTHFR genotype and blood pressure: Current evidence and implications for personalised nutrition. Proc. Nutr. Soc..

[38] Limveeraprajak N., Nakhawatchana S., Visukamol A., Siripakkaphant C., Suttajit S., Srisurapanont M. (2024). Efficacy and acceptability of S-adenosyl-L-methionine (SAMe) for depressed patients: A systematic review and meta-analysis. Prog. Neuropsychopharmacol. Biol. Psychiatry.

[39] Sarris J., Murphy J., Stough C., Mischoulon D., Bousman C., MacDonald P., Adams L., Nazareth S., Oliver G., Cribb L. (2020). S-Adenosylmethionine (SAMe) monotherapy for depression: An 8-week double-blind, randomised, controlled trial. Psychopharmacology.

[40] Bressa G.M. (1994). S-adenosyl-l-methionine (SAMe) as antidepressant: Meta-analysis of clinical studies. Acta Neurol. Scand. Suppl..

[41] Swardfager W., Herrmann N., Mazereeuw G., Goldberger K., Harimoto T., Lanctôt K.L. (2013). Zinc in depression: A meta-analysis. Biol. Psychiatry.

[42] Tarleton E.K., Littenberg B., MacLean C.D., Kennedy A.G., Daley C. (2017). Role of magnesium supplementation in the treatment of depression: A randomized clinical trial. PLoS ONE.

[43] Mocking R.J.T., Harmsen I., Assies J., Koeter M.W.J., Ruhé H.G., Schene A.H. (2016). Meta-analysis and meta-regression of omega-3 polyunsaturated fatty acid supplementation for major depressive disorder. Transl. Psychiatry.

[44] Dean O., Giorlando F., Berk M. (2011). N-acetylcysteine in psychiatry: Current therapeutic evidence and potential mechanisms of action. J. Psychiatry Neurosci..

[45] Sarris J., Logan A.C., Akbaraly T.N., Amminger G.P., Balanzá-Martínez V., Freeman M.P., Hibbeln J., Matsuoka Y., Mischoulon D., Mizoue T. (2015). Nutritional medicine as mainstream in psychiatry. Lancet Psychiatry.

[46] Rainka M., Meaney J., Westphal E.S., Aladeen T., Landolf K., Stanford S., Galdun P., Asbach N., Gengo F., Capote H. (2019). Effect of L-methylfolate on depressive symptoms in patients with MTHFR mutations (P3.9-057). Neurology.

[47] Meena R. (2025). Major Depressive Disorder (MDD), Pharmacogenomics, Antidepressant Therapy, Personalized Medicine, CYP2D6, CYP2C19. J. Pharmacol. Genet. Mol. Biol..

[48] Kessler R.C., Andrews G., Colpe L.J., Hiripi E., Mroczek D.K., Normand S.L.T., Walters E.E., Zaslavsky A.M. (2002). Short screening scales to monitor population prevalences and trends in non-specific psychological distress. Psychol. Med..

[49] R Core Team (2024). R: A Language and Environment for Statistical Computing.

[50] Fox J., Weisberg S. (2019). An R Companion to Applied Regression.

[51] Torchiano M. (2020). R Package.

[52] Champely S. (2020). R Package.

[53] Faul F., Erdfelder E., Lang A.-G., Buchner A. (2007). G*Power 3: A flexible statistical power analysis program for the social, behavioral, and biomedical sciences. Behav. Res. Methods.

[54] Fryar-Williams S. (2016). Fundamental role of methylenetetrahydrofolate reductase 677 C → T genotype and flavin compounds in biochemical phenotypes for schizophrenia and schizoaffective psychosis. Front. Psychiatry.

[55] Galizia I., Oldani L., Macritchie K., Amari E., Dougall D., Jones T.N., Lam R.W., Massei G.J., Yatham L.N., Young A.H. (2016). S-adenosyl methionine (SAMe) for depression in adults. Cochrane Database Syst. Rev..

