# Supplementary material for: Personalised Nutraceutical Treatment Guided by MTHFR Genotype in Mental Health: A Retrospective Cohort Study
Source: Nutrients. 2026 Aug 26;18(17):2791. doi: 10.3390/nu18172791 (PMC13566919; doi:10.3390/nu18172791)
Supplement: Supplementary file 1 [file nutrients-18-02791-s001.zip › nutrients-4493618-supplementary.pdf]

Supplementary Table S1: Patient *MTHFR* genotype, baseline biomarker profile, and treatment/supplement regimen

**Patient**	***MTHFR* genotype**	**Influencing Biomarkers (normal ref range in brackets)**				**Treatment provided (daily dose, where available)**	
		**Homocysteine (3-12)**	**Vitamin B12 (>150)**	**Serum folate (>7.0)**	**Serum zinc (10-25) or plasma zinc (9.0-19.0)**	**Concomitant medications**	**Supplements**
1	c.[665=];[665C>T]<br>c.[1286=];[1286A>C]	10.1	115	37	10.9 (plasma)	Sertraline 100 mg<br><br>Melatonin 3-6 mg	Betaine anhydrous 1.8g; folate 5mg DFE; vitamin B12 3mg; Vitamin B2 90mg; Vitamin B6 45mg x3<br>Potassium iodide 376mg x2<br><br>Vitamin D3 (cholecalciferol 25 mcg) and Vitamin K2 (MK-7 45mcg) x4<br><br>Zinc citrate dihydrate 95.8mg<br><br>Niacinamide 500mg x2<br>SAMe 200 mg<br><br>Biotin 80mcg; choline 14mg; folate 334mcg DFE; vit B1 50mg; vit B12 100mcg; vit B2 3.6mg; riboflavin

							25mg; vit B3 80mg; vit B5 250mg; vit B6 25mg
2	c.[665=];[665=]<br>c.[1286A>C];[1286A>C]						Zinc picolinate 30 mg<br><br>Betaine anhydrous 1.8g; folate 5mg DFE; vitamin B12 3mg; Vitamin B2 90mg; Vitamin B6 45mg x2<br>Potassium iodide 376mg x2
3	c.[665=];[665=]<br>c.[1286A>C];[1286A>C]	5.7	429	40	11.6 (plasma)		SAMe 200 mg<br><br>Betaine anhydrous 1.8g; folate 5mg DFE; vitamin B12 3mg; Vitamin B2 90mg; Vitamin B6 45mg x2<br>Zinc picolinate 30 mg
4	c.[665=];[665=]<br>c.[1286=];[1286A>C]		1118	29.5	15		Vitamin B6 50 mg<br><br>Zinc picolinate 50 mg
5	c.[665=];[665C>T]<br>c.[1286=];[1286=]	7.6	451	49.8	11	Escitalopram 10mg	Betaine anhydrous 1.8g; folate 5mg DFE; vitamin B12 3mg; Vitamin B2 90mg; Vitamin B6 45mg x2<br><br>Chromium glycinate 53mg<br><br>Zinc amino acid chelate 10mg

							Iron bisglycinate 25mg
6	NA	8.8	1055	1511 (redcell)	10	Diazepam 5mg occasionally<br><br>Mirtazapine 15 mg	Magnesium glycinate 3g<br><br>5-hydroxytryptophan 100mg
7	c.[665=];[665=]<br>c.[1286=];[1286=]	7.9	957	43.1	13		Betaine anhydrous 1.8g; folate 5mg DFE; vitamin B12 3mg; Vitamin B2 90mg; Vitamin B6 45mg x2
8	c.[665=];[665C>T]<br>c.[1286=];[1286=]	9.3	407	51.6	13		Betaine anhydrous 1.8g; folate 5mg DFE; vitamin B12 3mg; Vitamin B2 90mg; Vitamin B6 45mg x2<br><br>St John’s wort 250mg
9	NA	10	205	31.8	13	Fluoxetine 20 mg	Betaine anhydrous 1.8g; folate 5mg DFE; vitamin B12 3mg; Vitamin B2 90mg; Vitamin B6 45mg x2<br><br>5-hydroxytryptophan 100mg<br>Zinc picolinate 15 mg<br><br>GABA (gamma-aminobutyric acid) 100mg<br>St John’s wort 250mg

10	c.[665C>T];[665C>T]<br>c.[1286=];[1286=]	13.3	320	26.7	13		Mecobalamin (co-methylcobalamin) (active B12) 500 mcg<br><br>Thiamin 100mg; riboflavin 12.7mg; niacin 108mg; vitamin B6 16.7mg; folate 667mcg; vitamin B12 400mcg; biotin 400mcg<br><br>St John's wort 250mg<br><br>Iron bisglycinate 25mg<br>Zinc picolinate 15 mg<br>Magnesium, dose not recorded
11	c.[665C>T];[665C>T]<br>c.[1286=];[1286=]	10.5	378	30.4	12	Melatonin 2 mg	Magnesium glycinate 120mg x2<br><br>Betaine anhydrous 1.8g; folate 5mg DFE; vitamin B12 3mg; Vitamin B2 90mg; Vitamin B6 45mg x2<br><br>Chromium glycinate 53mg
12	NA		356	>54.0		Diazepam 5 mg for anxious days	Potassium iodide 376mg x2<br><br>Betaine anhydrous 1.8g; folate 5mg DFE; vitamin B12 3mg; Vitamin B2 90mg; Vitamin B6 45mg x2

13	c.[665=];[665C>T]<br>c.[1286=];[1286=]	9.4	195	>54.0	13		Chromium glycinate 53mg
14	NA	8.1	425	>54.0	14		P5P 53.7 mg<br><br>Thiamin 100mg; riboflavin 12.7mg; niacin 108mg; vitamin B6 16.7mg; folate 667mcg; vitamin B12 400mcg; biotin 400mcg<br><br>Omega-3 (EPA/DHA), dose not recorded<br>Zinc picolinate 15 mg<br><br>Magnesium glycinate dihydrate 1g; Magnesium glycinate 1.3 g<br>SAMe 200 mg
15	c.[665=];[665C>T]<br>c.[1286=];[1286A>C]	9.8	908	>54.0	13		Vitamin B6 15mg; folate 1360mcg; vitamin B12 1000mcg<br><br>Biotin 80mcg; choline 14mg; folate 334mcg DFE; vit B1 50mg; vit B12 100mcg; vit B2 3.6mg; riboflavin 25mg; vit B3 80mg; vit B5 250mg; vit B6 25mg<br><br>Vitamin D3 (cholecalciferol 25 mcg); Vitamin K2 (MK-7 45mcg) x2

							Mecobalamin (co-methylcobalamin) (active B12) 500 mcg x2<br>Omega-3 1500 mg<br>Zinc picolinate 30 mg
16	c.[665=];[665C>T]<br>c.[1286=];[1286A>C]		476	34.8	11		
17	NA	15.6	266	29.5	12	Sertraline 50-100 mg; diazepam 5mg on occasion	5-hydroxytryptophan 100mg
18	c.[665C>T];[665C>T]<br>c.[1286=];[1286=]	16.2	146	14			Betaine anhydrous 1.8g; folate 5mg DFE; vitamin B12 3mg; Vitamin B2 90mg; Vitamin B6 45mg x2<br>Zinc picolinate 15 mg<br>SAMe 200mg
19	NA	14	174	36.7	13	Mirtazapine 15 mg	Potassium iodide 376mg x2<br>5-hydroxytryptophan 100mg<br>Zinc citrate dihydrate 95.8mg<br>P5P 53.7 mg<br>Magnesium glycinate 120mg x2

20	c.[665=];[665C>T] c.[1286=];[1286=]	11.4	494	44.1	12	Diazepam 5mg occasionally	Betaine anhydrous 1.8g; folate 5mg DFE; vitamin B12 3mg; Vitamin B2 90mg; Vitamin B6 45mg Zinc picolinate 30 mg<br><br>Omega-3 (EPA/DHA), dose not recorded<br>Magnesium, dose not recorded<br><br>Potassium iodide 376mg x2<br><br>Nicotinamide 500mg
21	c.[665=];[665C>T] c.[1286=];[1286A>C]	11.6	287	>54.0	11	Escitalopram 20 mg; oxazepam, dose not recorded	SAMe 200 mg<br><br>Thiamin 100mg; riboflavin 12.7mg; niacin 108mg; vitamin B6 16.7mg; folate 667mcg; vitamin B12 400mcg; biotin 400mcg<br><br>P5P 53.7 mg
22	c.[665=];[665C>T] c.[1286=];[1286A>C]	15	979	>54.0	13	Escitalopram 10 mg; quetiapine 25 mg	Chromium glycinate 53mg<br><br>Betaine anhydrous 1.8g; folate 5mg DFE; vitamin B12 3mg; Vitamin B2 90mg; Vitamin B6 45mg x3

							Magnesium orotate dihydrate 200mg; magnesium gluconate 92.76mg; magnesium amino acid chelate 275mg; magnesium phosphate pentahydrate 115mg
23	NA	6.2	391	47.8	10	Escitalopram 10 mg	Potassium iodide 376mg x2<br><br>5-hydroxytryptophan 100mg<br>Zinc glycinate 40.97mg; zinc citrate dihydrate 38.94mg; vitamin B6 15mg<br><br>Zinc picolinate 30 mg<br>Methylfolate/B12 400 mcg
24	c.[665=];[665=]<br>c.[1286=];[1286A>C]	13.6					St John's wort 250mg
25	c.[665=];[665C>T]<br>c.[1286=];[1286A>C]	9.3	587	>54.0	12		Betaine anhydrous 1.8g; folate 5mg DFE; vitamin B12 3mg; Vitamin B2 90mg; Vitamin B6 45mg x2
26	c.[665=];[665=]<br>c.[1286=];[1286A>C]				8		Vitamin B6 50 mg; zinc picolinate 50 mg<br><br>Thiamin 100mg; riboflavin 12.7mg; niacin 108mg; vitamin B6 16.7mg;

							folate 667mcg; vitamin B12 400mcg; biotin 400mcg
27	NA	13.2	460	33.3	13	Diazepam 5 mg; sertraline 50mg	GABA (gamma-aminobutyric acid) 100mg<br><br>5-hydroxytryptophan 100mg<br>St John’s wort 250mg
28	NA					Diazepam 5mg occasionally	Chromium glycinate 53mg<br><br>Zinc amino acid chelate 10mg<br><br>Iron bisglycinate 25mg x1 every second day<br>Vitamin D3 5000 IU 3 days/week<br><br>5-hydroxytryptophan 150mg
29	c.[665=];[665C>T]<br>c.[1286=];[1286=]	5.7	325	20	12.2 (plasma)	Escitalopram 10mg; Dexamphetamine 5 mg; Inderal 10 mg; Sertraline 50mg	Biotin 80mcg; choline 14mg; folate 334mcg DFE; vit B1 50mg; vit B12 100mcg; vit B2 3.6mg; riboflavin 25mg; vit B3 80mg; vit B5 250mg; vit B6 25mg<br><br>Zinc citrate dihydrate 95.8mg<br><br>Magnesium glycinate 120mg x2

30	c.[665=];[665C>T]<br>c.[1286=];[1286A>C]		421	36	14	Lisdexamfetamine 30 mg; dexamphetamine 5mg	Vitamin D3 (cholecalciferol 25 mcg) and Vitamin K2 (MK-7 45mcg) x3<br><br>Zinc picolinate 15 mg<br><br>Betaine anhydrous 1.8g; folate 5mg DFE; vitamin B12 3mg; Vitamin B2 90mg; Vitamin B6 45mg x2<br>St John's wort 250mg
31	c.[665=];[665C>T]<br>c.[1286=];[1286=]	20.2	370	22.4	13	Dexamphetamine 5 mg x2	SAMe 200 mg<br><br>Biotin 80mcg; choline 14mg; folate 334mcg DFE; vit B1 50mg; vit B12 100mcg; vit B2 3.6mg; riboflavin 25mg; vit B3 80mg; vit B5 250mg; vit B6 25mg<br>x2<br><br>Zinc picolinate 15 mg
32	NA	8.4	648	>54.0	19		
33	NA		772	>54.0	13	Naltrexone 4.5 mg; Lithium orotate 5 mg	Magnesium citrate, dose not recorded
34	c.[665=];[665C>T]<br>c.[1286=];[1286=]	10.7	631	>54.0	10	Lithium orotate 4 mg	Zinc picolinate 15 mg<br>Potassium iodide 376mg x3

							Magnesium glycinate dihydrate 1g; Magnesium glycinate 1.3g; vitamin B5 50mg; vitamin B1 38mg; vitamin B6 25mg; vitamin B3 50mg; vitamin B2 25mg
35	c.[665C>T];[665C>T]<br>c.[1286=];[1286=]				12.1 (plasma)		SAMe 200 mg<br><br>Thiamin 100mg; riboflavin 12.7mg; niacin 108mg; vitamin B6 16.7mg; folate 667mcg; vitamin B12 400mcg; biotin 400mcg
36	NA	7.3	455	19	12.5 (plasma)	Escitalopram 10mg	Potassium iodide 376mg x2<br><br>Zinc picolinate 15 mg<br><br>Betaine anhydrous 1.8g; folate 5mg DFE; vitamin B12 3mg; Vitamin B2 90mg; Vitamin B6 45mg<br><br>Mecobalamin (co-methylcobalamin) (active B12) 500 mcg

							GABA (gamma-aminobutyric acid) 100mg<br><br>Magnesium glycinate dihydrate 1g; Magnesium glycinate 1.3g; vitamin B5 50mg; vitamin B1 38mg; vitamin B6 25mg; vitamin B3 50mg; vitamin B2 25mg
37	NA	10.3	238	33.9	14	Sertraline 50mg	Betaine anhydrous 1.8g; folate 5mg DFE; vitamin B12 3mg; Vitamin B2 90mg; Vitamin B6 45mg x2
38	c.[665=];[665C>T]<br>c.[1286=];[1286A>C]	8.7	415	40.4	12	Oxazepam 15 mg	GABA (gamma-aminobutyric acid) 100mg<br><br>Magnesium glycinate 120mg
39	c.[665=];[665C>T]<br>c.[1286=];[1286=]	15	446	27.8	11	Lisdexamfetamine 70 mg	Betaine anhydrous 1.8g; folate 5mg DFE; vitamin B12 3mg; Vitamin B2 90mg; Vitamin B6 45mg<br><br>Zinc picolinate 15 mg<br><br>Magnesium glycinate 120mg x2

40	c.[665=];[665C>T]<br>c.[1286=];[1286A>C]	6.3	500	30.3	11	Vortioxetine hydrobromide 10 mg	Magnesium amino acid chelate 1050mg; magnesium glycinate dihydrate 427.35mg; magnesium citrate 323.6mg<br>Zinc picolinate 30 mg
41	c.[665=];[665C>T]<br>c.[1286=];[1286=]	13.9	275	17.7	12	Escitalopram 10mg	Zinc picolinate 15 mg<br><br>Betaine anhydrous 1.8g; folate 5mg DFE; vitamin B12 3mg; Vitamin B2 90mg; Vitamin B6 45mg
42	c.[665=];[665=]<br>c.[1286=];[1286=]	9.5	365	31	15.9 (plasma)	Mirtazapine 15 mg; Fluoxetine 20 mg; Mianserin 10mg	Magnesium glycinate 120mg x2
43	c.[665=];[665=]<br>c.[1286A>C];[1286A>C]		422	23		Fluoxetine 15mg	P5P 53.7 mg
44	c.[665=];[665C>T]<br>c.[1286=];[1286A>C]	7.7	221	34	9.8 (plasma)	Zopiclone 7.5 mg	GABA (gamma-aminobutyric acid) 100mg<br>EPA 500 mg; DHA 200 mg
45	c.[665=];[665C>T]<br>c.[1286=];[1286=]	5.2	292	18	12.9 (plasma)		Vitamin B6 50 mg; zinc picolinate 50 mg
46	c.[665=];[665C>T]<br>c.[1286=];[1286=]	13.5	426	23.9	15		L-theanine 100 mg

47	c.[665=];[665C>T]<br>c.[1286=];[1286A>C]	8.7	219	19	12.8 (plasma)	Lithium orotate 1125mg; Vortioxetine hydrobromide 30 mg; Olanzapine 2.5 mg	
48	c.[665=];[665C>T]<br>c.[1286=];[1286A>C]	11.9				Sertraline 25 mg	Zinc picolinate 30 mg<br><br>5-hydroxytryptophan 100mg
49	c.[665=];[665=]<br>c.[1286=];[1286A>C]	10.5	248	34.6	9	Sertraline 100mg	
50	c.[665=];[665C>T]<br>c.[1286=];[1286=]	4.9			13.4		Magnesium (unspecified), dose not recorded<br>Zinc picolinate 15 mg<br>Vitamin B formulation, dose not recorded

Supplementary Table S2. Missingness sensitivity analysis for paired biomarker follow-up

Comparison of baseline characteristics between patients with vs. without paired follow-up biomarker data. Age and baseline K10 compared by Welch two-sample t-test; sex compared by Fisher's exact test.

**Biomarker**	**Group**	**n**	**Age (mean ± SD)**	**Female (%)**	**Baseline K10 (mean ± SD)**	**p (age)**	**p (sex)**	**p (baseline K10)**
Homocysteine (n=30)	Paired (retested)	30	45.5 ± 16.1	76.7%	23.3 ± 9.6	0.063	0.033	0.449
	Unpaired (not retested)	20	38.5 ± 9.9	100.0%	25.7 ± 11.4			
B12 (n=37)	Paired (retested)	37	44.0 ± 15.1	81.1%	24.6 ± 9.0	0.204	0.168	0.733
	Unpaired (not retested)	13	38.9 ± 11.0	100.0%	23.2 ± 13.7			
Serum folate	Paired (retested)	19	42.9 ± 17.0	78.9%	26.2 ± 10.8	0.930	0.404	0.316
	Unpaired (not retested)	31	42.5 ± 12.6	90.3%	23.1 ± 10.0			

Legend. Sensitivity analyses compare baseline characteristics between patients with paired follow-up biomarker data (i.e., contributed to paired biomarker–K10 correlations) and those without. Homocysteine paired-vs-unpaired sex distribution reached nominal significance (Fisher $p = 0.033$); no other differences reached $p < 0.05$. Baseline K10 and age did not differ meaningfully between groups for any biomarker, indicating no evidence of informative missingness on the primary outcome distribution.
